# On the Treatment of Whooping Cough by Diluted Nitric Acid

**Published:** 1860-07

**Authors:** John Atcherley

**Affiliations:** M.R.C.S., Eng.


### ON THE TREATMENT OF WHOOPING-COUGH BY DILUTED NITRIC ACID.

BY JOHN ATCHERLEY, M.R.C.S., ENG.

I wish to direct the attention of the profession to the diluted nitric acid in the treatment of whooping-cough. It has already been employed in whooping-cough to some extent, and was first recommended some years ago, by Dr. Arnold, of Montreal.

Having had abundant opportunities during the last two years, of testing its efficacy, I may be permitted to speak with some degree of confidence as to its value. I have confined myself exclusively to its use in every case I have had to treat throughout that period, and I can affirm as to the ordinary duration of whooping-cough has been computed to average ten weeks, in defiance of every method of treatment that had hitherto been adopted, the diluted nitric acid effects its removal in less than three weeks, except in cases where its course had been interrupted by some serious complications.

Any medicine capable of abridging the duration of a disease whose fatality is in proportion to its continuance, must be of incalculable value. I am convinced that when it becomes more generally used, it will meet with the concurrence of the profession, and will hold a high place, and be the cardinal remedy, if not supercede all other medicines in whooping cough.

In prescribing the diluted nitric acid, I usually begin with five minim doses every three hours, say for a child six months old, and gradually increase the dose, in proportion to the age, to fifteen minims every second hour, should the paroxysms become aggravated, or of more frequent recurrence. When the intervals become lengthened, which generally happens after the second day, the medicine may be given less frequently; but it is of importance that the acid should be continued ten days after all symptoms of the disease have subsided. From the neglect of this precaution, I have seen the cough return with all its former violence when the medicine has been abruptly discontinued; therefore, it should be given in moderate doses, three times a day, after all traces of the affection have passed away.

The form I generally use is the one originally suggested, viz: diluted nitric acid, compound tincture of cardamoms, syrup and water. This is always taken without the slightest reluctance, as it is agreeable to the taste—a great consideration in prescribing medicine for children, which requires to be continued for some length of time.

In conjunction with the above treatment, I have invariably employed a stimulating embrocation to the back and chest, night and morning, consisting of one ounce of camphor liniment, and two drachms of spirits turpentine.

Of course, it is necessary as in all diseases of the respiratory organs, that proper attention should be paid to the state of the bowels, regulation of temperature, clothing and diet. I have also seen great benefit derived from the inhalation of the fumes of burning nitre-paper; two pieces of about four inches square are burnt in the bed-room on retiring to rest, and one piece burnt occasionally in the room occupied by the child in the day-time, appears to shorten the paroxysm, and to deprive it in a great measure of its spasmodic character, rendering it more like the cough of ordinary catarrh. Chloroform is the best anti-spasmodic that can be used during the fit, but parents have a great dread of its effects, unless administered by the medical attendant; but from the apparent simplicity in the fumes of burning nitre-paper, they are readily induced to give it a trial.—*Med. Times and Gazette.*

